# Effects of Sonication and Thermal Pasteurization on the Nutritional, Antioxidant, and Microbial Properties of Noni Juice

**DOI:** 10.3390/molecules28010313

**Published:** 2022-12-30

**Authors:** Yi Xuan Choo, Lai Kuan Teh, Chin Xuan Tan

**Affiliations:** Department of Allied Health Sciences, Faculty of Science, Universiti Tunku Abdul Rahman, Kampar Campus, Kampar 31900, Perak, Malaysia

**Keywords:** sonication, juice, noni, *Morinda citrifolia* L.

## Abstract

Sonication is recognized as a potential food processing method to improve the functional properties of fruit juice. This study evaluated the effects of different sonication durations (20, 40, and 60 min) and thermal pasteurization on the nutritional, antioxidant, and microbial properties of noni juice. Fresh noni juice served as the control. The main organic acids detected were malic (57.54–89.31 mg/100 mL) and ascorbic (17.15–31.55 mg/100 mL) acids. Compared with the fresh sample, the concentrations of these compounds were significantly improved (*p* < 0.05) in the 60 min sonicated sample but reduced (*p* < 0.05) in the pasteurized sample. Moreover, sonication for 60 min resulted in increments of scopoletin, rutin, and vanillic acid compared to the fresh sample. The antioxidant activity of the juice sample was improved in the sample sonicated for 60 min. Irrespective of juice processing method, the level of microbial counts in noni juice was within the satisfactory level over the 8 weeks of refrigerated (4 °C) storage. This study highlights the feasibility of using ultrasound processing to enhance the quality of noni juice on the industrial scale.

## 1. Introduction

Noni (*Morinda citrifolia* L.), also known as Indian mulberry and mengkudu, is an evergreen plant species belonging to the *Rubiaceae* family. The plant bears fruits throughout the year and is widely distributed in tropical and subtropical regions. Noni fruit is oval, 4–10 cm in length, 3–8 cm in circumference, and 50–300 g in weight and contains numerous small drupes fused to its rough surface [1]. Ripe noni fruit is soft and translucent–greyish and contains approximately 90% water [1]. The fruit has been used as a medicine for the treatment of an array of non-communicable diseases [2].

In the early 1990s, the first commercialized noni product was launched in the USA [3]. Since then, countless products derived from noni fruit, such as juice, puree, powder, gel capsules, extract, syrup, pills, and lozenges, have been introduced to the market. The annual sales of noni products are expected to reach up to USD 1.3 billion [3]. Noni juice has a significant presence in the functional beverage market [4]. The functional beverage market is the largest and fastest-growing segment of the functional food sector [5]. Functional beverages are rich in phytonutrients, which could provide benefits to human health beyond their nutritional value. However, the levels of phytonutrients could be affected by food processing methods. Traditionally, noni juice is produced through fermentation of the fruits in a sealed barrel for 10 to 60 days. An uncontrolled fermentation process may result in the formation of a large amount of alcohol in the noni juice [6]. This affects the organoleptic characteristics of the juice. Other manufacturing techniques of noni juice are direct hand- pressing and machine extraction, such as with a juice extractor or centrifuge [4]. Irrespective of the extraction method, raw noni juice is usually subjected to thermal pasteurization to eliminate the harmful microbes that might be present [7].

Pasteurization is a thermal processing technique that hinders the action of microorganisms and enzymes [8], assuring the safety of juice. High temperatures used in pasteurization might affect the physicochemical properties of juice [9]. Alternative food processing techniques have been sought to overcome the potentially detrimental effects of thermal pasteurization on fruit juice. Sonication of fruit juice has received considerable attention in recent years. It has shown the potential to fulfill the Food and Drug Administration (FDA) requirement of a five-log reduction in relevant microorganisms found in fruit juice yet retaining or even enhancing the nutritional properties of the fruit juice [8,10]. Previously, sonication was reported as a good alternative to thermal pasteurization of barberry juice [10], pomelo juice [8], and elephant apple juice [9]. To the best of our knowledge, there are no or few studies investigating the effect of sonication processing on the quality of noni juice. Hence, the objective of this study is to determine the influence of ultrasound processing on the nutritional, antioxidant, and microbial properties of noni juice and to compare the obtained results with fresh and thermal pasteurized noni juice samples.

## 2. Results and Discussion

### 2.1. Total Soluble Solids, pH, Titratable Acidity, Viscosity, and Color

Total soluble solids measures the level of sweetness of fruit juice. The TSS of noni juice was found to be in the range of 1.30–1.37 °Brix, and the levels were unaffected by the juice processing method and sonication duration (Table 1). A higher level of TSS (5.8 °Brix) was reported in enzyme-treated hydraulically pressed noni juice [2], possibly due to the release of more sugars and organic acids following the tissue breakdown by enzymes. Noni pulp is bitter or astringent rather than sweet [4]. The TSS of noni juice is lower than that of apple (11.5 °Brix), banana (22.0 °Brix), grape (16.0 °Brix), orange (11.8 °Brix), and pomegranate (16.0 °Brix) juices [11].

The pH and titratable acidity of noni juice ranged from 3.90 to 3.91 and 0.17 to 0.18%, respectively. No differences were found in the pH and titratable acidity of fresh, sonicated, and pasteurized juice samples. Chitgar et al. [10] also observed no differences in pH and titratable acidity of fresh, sonicated, and pasteurized barberry juice. Fruit juices with pH values less than 4.6 are deemed highly acidic [12]. The pH value obtained in the present study was comparable with that of fermented noni juice (pH = 3.7) [13], indicating the acidic nature of this juice. According to Nowak et al. [14], the low pH value in noni juice was associated with its high ascorbic acid content.

On the other hand, noni juices with sonication times of 40 min (S40) and 60 min (S60) were significantly more viscous (*p* < 0.05) than fresh noni juice. No significant difference (*p* > 0.05) was observed between the viscosity level of S20 and fresh noni juice. These results suggest that more soluble pectin might leach from the cell walls of noni fruit following a longer sonication duration. Hence, more concentrated pectin colloidal juices were obtained in the S40 and S60 samples. Likewise, Abid et al. [15] observed that the viscosity levels of apple juice following more than 30 min sonication were significantly greater than those of non-sonicated sample. The present study demonstrated that the viscosity level of pasteurized noni juice (14.40 mPa S) was significantly lower (*p* < 0.05) than that of fresh (16.27 mPa S) and all sonicated (17.07–17.33 mPa S) noni juices. When noni juice is heated, the viscosity reduces as the thermal energy of the molecules increases, and the intermolecular distances become greater due to thermal expansion [16].

The color of fruit juice serves as the basis for consumers to judge the overall product quality. As shown in Table 1, sonication resulted in significant differences (*p* < 0.05) in the lightness (L*), yellowness (+b*), and chroma (C*) values of noni juice as compared with fresh juice. The results of the present study are in agreement with the findings of Santhirasegaram et al. [17], who reported that sonicated mango juice exhibited an increased L* value and a reduced +b* value. The color of the noni juice became less saturated, as indicated by reduced C* values after sonication. The hue angle (*h°*) of noni juice was unaffected by the juice processing method. There were no differences (*p* > 0.05) in the three chromaticity coordinates (L*, a*, and b* values) of pasteurized and fresh noni juice, in contrast to a study on barberry juice [10]. Manzoor et al. [18] reported no difference in L* and a* values between pasteurized and fresh sugarcane juice, but the b* value was reduced after pasteurization. Generally, all three chromaticity coordinates in juice are greatly affected by the conditions of food processing and the composition of the fruit cultivars [18,19].

Total color difference (Δ*E*) is a useful measure to determine the differences in perceivable color. It can be classified as very distinct (Δ*E* > 3), distinct (1.5 < Δ*E* < 3), and small difference (Δ*E* < 1.5) [18]. Sonication (Δ*E* 5.20–6.11) resulted in a very distinct color variation in comparison to the fresh noni juice, possibly as a result of ultrasound-induced hydroxylation of the phenolic aromatic ring, which, in turn, changes the visible spectrum area [18]. Consistent with the findings of Manzoor et al. [18], pasteurization (Δ*E* 1.16) resulted in a small color difference in the juice.

### 2.2. Total Carotenoids, Phenolics, and Flavonoids

Carotenoids correspond to the red, yellow, or orange pigment of fruits. In this study, the carotenoid content of noni juice was measured spectrophotometrically using *β*-carotene as a standard. Table 2 shows the TC, TPC, and TFC of noni juice samples. A relatively low level of carotenoid (<0.2 mg *β*CE/100 mL) was observed in the noni juice, with no significant difference (*p* > 0.05) between fresh, pasteurized, and sonicated (S20, S40, and S60) samples. Barraza-Elenes et al. [13] also reported a low level of carotenoid content (1.06 mg *β*CE/100 g) in noni juice. The levels of carotenoids in fruits can be predicted by their color [20]. Hence, translucent–greyish noni fruit is expected to be low in carotenoids. Additionally, the lipophilic nature of carotenoids might make these compounds poorly leach out from the fruit during juice extraction.

More than 150 phytochemicals have been detected in noni fruit. Among these, phenolics are the main phytochemicals [21]. Phenolics are one of the main contributors to antioxidant activity in fruit juice. In the present study, the TPC ranged from 2.62 to 3.19 mg GAE/100 mL for noni juice samples. There is a large variation in the TPC of noni juice reported in the literature, ranging from 3.1 to 210 mg GAE/100 mL [22,23,24]. Although Folin–Ciocalteu colorimetric assay is commonly used to gauge the phenolic content of beverages, the difference in the sample preparation parameters (e.g., extraction solvents, incubation time, etc.) could greatly influence the TPC results [25]. Our study indicated that the TPC of noni juice subjected to 60 min sonication (3.19 mg GAE/100 mL) was significantly greater than (*p* < 0.05) that of pasteurized, fresh, and other sonicated noni juice samples (2.62–2.93 mg GAE/100 mL). This is in agreement with the findings of Bhat et al. [26], who reported that the TPC of lime juice significantly improved after 60 min of sonication.

Flavonoids are natural phenolic compounds present in fruits with anti-inflammatory properties. As shown in Table 2, the TFC of noni juice ranged from 1.01 to 1.48 mg RE/100 mL. These values are in accordance with the TFC values (0.66–2.48 mg quercetin equivalents/100 mL) of fermented noni juice [23]. The current study demonstrated that sonication of the noni juice for 60 min significantly increased (*p* < 0.05) the yield of TFC by 47%, as compared with the fresh sample. Following 60 min of sonication, the increment of TPC and TFC in the noni juice might be related to the liberation of bound phenolics and flavonoids. It might be also attributable to the attachment of sonochemically generated hydroxyl radicals to the aromatic rings of phenolic and flavonoid compounds [26].

High-performance liquid chromatography was used to quantify the selected phenolic compounds in the noni juice. Vanillic acid (7.95–12.17 mg/100 mL), rutin (2.22–4.02 mg/100 mL), and scopoletin (0.83–1.47 mg/100 mL) were detected in the fresh, sonicated (S20, S40, and S60), and pasteurized noni juice samples (Table 3). In contrast with the study by Deng et al. [27], in the current study, we did not detect the presence of quercetin in noni juice, possibly due to the use of different wavelengths in HPLC UV detection. The noni juice subjected to 60 min sonication yielded significantly (*p* < 0.05) more scopoletin, rutin, and vanillic acid than the fresh and pasteurized samples. These observations correspond to the TPC results presented in Table 2. Compared with the fresh sample, sonication for 60 min resulted in an increment of 53% of scopoletin, 46% of rutin, and 35% of vanillic acid in the noni juice. A similar observation was reported by Abid et al. [15], where the individual phenolic compounds in the apple juice significantly increased after 60 min sonication. However, no significant difference (*p* > 0.05) was observed between the scopoletin and rutin levels of the fresh, S20 and S40 samples, whereas the vanillic acid level showed otherwise. This might be attributable to incomplete cell wall disruption due to an insufficient sonication; hence, fewer bound phenolics were liberated.

### 2.3. Organic Acids

Organic acids are the second most abundant soluble solids in fruit juice. Four organic acids, namely malic (57.54–89.31 mg/100 mL), ascorbic (17.15–31.55 mg/100 mL), citric (0.90–4.78 mg/100 mL), and fumaric (0.35–0.51 mg/100 mL) acids were identified in noni juice (Table 3). Total organic acids were detected in the order of S60 (126.09 mg/100 mL) > S40 (119.54 mg/100 mL) > S20 (114.62 mg/100 mL) > FRE (105.25 mg/100 mL) > PAS (75.94 mg/100 mL).

The present study indicated malic acid as the predominant organic acid in noni juice. Bittová et al. [28] also reported malic acid as the main organic compound in commercial noni fruit products, such as powder, capsules, and juice. This organic acid was originally extracted from apple juice and has broad applications in food, pharmaceuticals, plastic production, and metal cleaning. Compared to fresh noni juice, sonication for 60 min significantly increased (*p* < 0.05) the yield of malic acid by 17%, whereas pasteurization significantly reduced (*p* < 0.05) the yield of malic acid by 25%. Giavoni et al. [29] observed that the malic acid of orange pulp byproduct reduced by 18%, from 74.78 to 61.08 mg/100 g, following pasteurization. Regardless of the juice processing method, the contents of citric and fumaric acids were found to be low in noni juice. Chunhieng et al. [30] also found a low amount of citric acid in hydraulically pressed noni juice (3 mg/100 mL). The citric and fumaric acids of noni juice were significantly improved (*p* < 0.05) after sonication for 40 and 60 min. Generally, longer sonication times might result in a greater mechanical rupture of the cell wall and intracellular structures, such as plastids. This promotes the release of these organic compounds into the aqueous medium of the beverage, leading their concentrations to increase [31].

Results of the current study indicated a significant increase (*p* < 0.05) in ascorbic acid in the noni juice sonicated for 40 and 60 min compared to the control (fresh juice). The same results were observed in sonicated apple, lime, and grapefruit juices [26,32,33]. These studies showed that the ascorbic acid level of fruit juices subjected to ultrasound processing for more than 30 min was enhanced as compared with control samples. The increase in ascorbic acid in noni juice could be due to the mild temperature used for sonication and the elimination of dissolved oxygen by cavitation. The main factors contribute to ascorbic acid degradation are heat and oxygen [31]. Moreover, pasteurization reduced 36% of the ascorbic acid content in the noni juice compared to the control because the high-heat processing used in pasteurization might result in the oxidation of ascorbic acid to dehydroascorbic acid.

### 2.4. Antioxidant Capacity

The antioxidant activity of noni juice was evaluated using FRAP and TEAC assays. The results are presented in Table 4. FRAP measures the ability of bioactive compounds in noni juice to reduce the colorless ferric tripyridyl triazine (Fe^3+^-TPTZ) to an intense blue-colored ferrous tripyridyltriazine (Fe^2+^-TPTZ) at pH 3.6. The FRAP values ranged from 29.92 to 59.63 µM Fe^2+^/kg. Noni juice subjected to 60 min sonication resulted in a significantly greater (*p* < 0.05) FRAP value than the fresh, pasteurized, and other sonicated juice samples. TEAC measures the ability of bioactive compounds in noni juice to inhibit the ABTS radical cation induced by potassium peroxodisulfate. The TEAC values of noni juice were in the range of 15.52–19.65 mM TE/kg. Noni juice subjected to 40 min (S40) and 60 min (S60) sonication had significantly greater (*p* < 0.05) TEAC values than the S20, fresh, and pasteurized samples.

Generally, the results of both assays demonstrated that 60 min sonication significantly improved (*p* < 0.05) the antioxidant capacity of noni juice compared to fresh juice. The increase in the antioxidant activity of S60 might be ascribed to the increase in bioactive compounds such as phenolics and organic acids (Table 2 and Table 3).

### 2.5. Microbial Activity

Food processing plays an important role in the inactivation of the naturally occurring microorganisms responsible for foodborne illness. Table 5 illustrates the microbial load (aerobic mesophilic bacteria, yeast, and mold) of fresh, pasteurized, and sonicated noni juices over 8 weeks of refrigerated (4 °C) storage. S60 was selected, as it contained the greatest amounts of phenolics, organic acids, and antioxidant capacity (Table 2, Table 3 and Table 4). Throughout the 8 weeks, yeast and mold were not detected, whereas the total number of aerobic mesophilic bacteria was maintained at less than 10^4^ CFU/mL, indicating that noni juice fell under the satisfactory level of microbiological standards for ready-to-eat food [34]. The use of noni juice in preserving the quality of fresh-cut mango cubes was demonstrated by Ulloa et al. [35]. In their study, the presence of antioxidant compounds such as phenolics was used to explain the antimicrobial activity of noni juice towards the mango cubes over 15 days of refrigerated storage. However, the present study showed no difference (*p* > 0.05) in the microbial counts (aerobic mesophilic bacteria, yeast, and mold) between the fresh and high-phenolic S60 samples after 8 weeks of storage. The antimicrobial properties of noni juice might be associated with its low pH (Table 1) because most of the microorganisms do not grow or grow very slowly at pH values lower than 4.6 [36]. In agreement with Basumatary et al. [8], pasteurization was found to be better than sonication in inactivating microorganisms of the fruit juice. The high temperatures used in the pasteurization process might destroy organic substances that are essential for the proper functioning of microbes, resulting in cell lysis. Irrespective of the juice processing method, the level of microbial counts in noni juice was within the satisfactory range.

## 3. Materials and Methods

### 3.1. Preparation of Noni Juice

Hard white noni fruit was harvested in October 2021 from Sureco Sure Return Farm, Perak Malaysia. The fruit was washed with tap water and sanitized with a bleach solution (1 teaspoon of bleach per 4 L of distilled water). The surface of the fruit was dried with absorbent tissue paper before being kept at room temperature (25 °C ± 2 °C) for 1–2 days to ripen, as characterized by translucent–greyish color and soft texture [13]. A stainless-steel knife was used to cut the fruit into halves, and the seeds were manually separated. The peel and the pulp were extracted using a commercial juice processor (Nippon, Selangor, Malaysia). The juice was filtered through a sterile muslin cloth and then equally divided into these five groups: fresh (FRE), sonication for 20 min (S20), sonication for 40 min (S40), sonication for 60 min (S60), and pasteurization (PAS). Each group had triplicates.

### 3.2. Sonication and Pasteurization Treatments

Noni juice was sonicated using an ultrasonic bath (Elmasonic EASY, Baden-Wurttemberg, Germany; 37 kHz ultrasonic frequency, 600 W ultrasonic output power, 11.8″ × 9.4″ × 5.9″ (L × W × H) internal dimensions) at different time intervals (20, 40, and 60 min) under a constant temperature of 30 °C and a frequency of 37 kHz [26]. To avoid light interference, the sonication process was conducted in a dark environment. Pasteurization of noni juice was accomplished according to the method of Chitgar et al. [10] under a temperature of 90 °C for 60 s. All juice samples were stored in brown bottles wrapped with aluminum foil at −20 °C until use. Fresh noni juice served as the negative control in this study.

### 3.3. Total Soluble Solids (TSS), pH, Titratable Acidity, Viscosity, and Color

The TSS was measured using a handheld refractometer (Atago PAL-3, Tokyo, Japan) at room temperature. A digital pH meter (Eutech pH 700, Waltham, MA, USA) was used to measure the pH of noni juice. The titratable acidity was determined using the AOAC 942.15 standard procedure [37]. A rotary viscometer (Brookfield DV2T, Berwyn, IL, USA) was used to measure the viscosity of noni juice. Color attributes were measured using a colorimeter (Konica Minolta CM-600d, Osaka, Japan). Results were expressed as L*, a*, and b*. L* measures luminosity/lightness on a scale of 0 (black) to 100 (white). a* indicates green when negative and red when positive, whereas the b* indicates blue when negative and yellow when positive. Chroma (C*), hue angle (*h°*), and the total color difference (Δ*E*) were calculated using the following equations:

(1) $$C^{*}=\sqrt{{\left(a^{*}\right)}^{2}+{\left(b^{*}\right)}^{2}}$$

(2) $$h^{{}^{\circ}}=180+\tan^{-1}\left(\frac{b^{*}}{a^{*}}\right),\;\mathrm{when}\;a^{*}<0\;$$

(3) $$\Delta E=\sqrt{\;{\left(\Delta L^{*}\right)}^{2}+{\left(\Delta \;a^{*}\right)}^{2}+{\left(\Delta b^{*}\right)}^{2}}\;$$

### 3.4. Sample Preparation for Total Carotenoids, Phenolics, Flavonoids, and Antioxidant Capacity

Exactly 1 mL of noni juice was pipetted into a test tube containing 5 mL of 60% methanol and centrifuged at 10,000 rpm for 15 min at room temperature. The supernatant layer (extract) was used for the determination of total carotenoids, phenolics, flavonoids, and antioxidant capacity (Trolox equivalent antioxidant capacity and ferric reducing antioxidant power) [38].

### 3.5. Total Phenolic Content (TPC)

The TPC was determined according to the method of Dars et al. [39]. Briefly, 100 μL of the juice extract was mixed with 400 μL of sterile ultrapure water and 500 μL of Folin–Ciocalteu reagent (1:10 *v*/*v*) in a falcon tube. After incubating in the dark for 5 min at room temperature, 1000 μL of 7.5% sodium carbonate solution was pipetted into the mixture. A falcon tube was incubated in the dark for 30 min at room temperature. A spectrophotometer (DLAB Scientific SP-V1000, Beijing, China) was used to measure the absorbance at 765 nm against a blank. The TPC was determined using a gallic acid standard curve, and the results were expressed as mg gallic acid equivalents (GAE)/100 mL of juice.

### 3.6. Total Flavonoid Content (TFC)

The TFC was analyzed according to the method of Abid et al. [32] with minor modifications. The extract (500 µL), sterile ultrapure water (250 µL), and 5% sodium nitrite solution (150 µL) were mixed and incubated at room temperature for 6 min. The mixture was then combined with 300 µL of 10% aluminum chloride solution. After 5 min, 1000 µL of 1 M sodium hydroxide solution was added. The absorbance was measured at 510 nm against a blank using a spectrophotometer (DLAB Scientific SP-V1000, Beijing, China). The TFC was determined using a rutin standard curve, and the results were expressed as mg rutin equivalents (RE)/100 mL of juice.

### 3.7. Total Carotenoid Content (TCC)

The TCC was determined according to the method of Tan et al. [40] with modifications. A calibration curve in the range of 0–2.5 µg/mL was initially constructed by dissolving the β-carotene in methanol. Exactly 250 µL extract was mixed with 500 µL methanol in a falcon tube and vortexed. The absorbance was measured at 440 nm against a blank using a spectrophotometer (DLAB Scientific SP-V1000, Beijing, China). The total carotenoid content of the sample was expressed as mg β-carotene equivalents (*β*CE)/100 mL juice.

### 3.8. Ferric Reducing Antioxidant Power (FRAP)

The FRAP was determined using the method described by Benzie and Devaki [41]. FRAP reagent was freshly prepared by combining 25 mL of 0.3 M acetate buffer (pH 3.6), 2.5 mL of 10 mM TPTZ solution in 40 mM hydrochloric acid, and 2.5 mL of 20 mM ferric chloride solution. The FRAP reagent (1.5 mL) was pipetted into a falcon tube containing 50 μL of extract and incubated at 37 °C for 10 min. The absorbance was measured against a blank at 593 nm using a spectrophotometer (DLAB Scientific SP-V1000, Beijing, China). A ferrous sulfate standard curve was constructed. The results were expressed as μM ferrous iron equivalents (Fe^2+^)/kg of juice.

### 3.9. Trolox Equivalent Antioxidant Capacity (TEAC)

The TEAC was determined according to the method of Tan et al. [40]. An ABTS radical cation stock solution was prepared by mixing 2.55 mM potassium peroxodisulfate with 7 mM ABTS powder in 10 mL deionized water in the dark for 16 h at room temperature. The working solution was prepared by diluting the stock solution with absolute ethanol to an absorbance of 0.70 ± 0.05 at 734 nm. Exactly 100 µL of the extract was mixed with 1000 µL of ABTS working solution and incubated at room temperature in the dark for 6 min. The absorbance of the mixture was measured against a blank at 734 nm using a spectrophotometer (DLAB Scientific SP-V1000, Beijing, China). A Trolox calibration curve was prepared. The TEAC of samples was expressed as mM of Trolox equivalent (TE) per kg of juice.

### 3.10. Sample Preparation for Quantification of Organic Acids and Phenolics

Approximately 5 mL of noni juice was centrifuged at 10,000 rpm for 15 min at room temperature. The supernatant layer was filtered through a 0.22 μm syringe filter, degassed at 25 °C for 5 min, and used for quantification of organic acids and phenolics.

### 3.11. Organic Acids

The quantification of organic acids in noni juice was performed according to the method of Scherer et al. [42]. A high-performance liquid chromatograph (HPLC) (Shimadzu LC-10AD, Kyoto, Japan) equipped a UV-Vis detector (Shimadzu SPD-20A, Kyoto, Japan) set at 210 nm was used. Exactly 20 μL of the filtered sample was injected into a LiChrospher RP-18 column (125 mm × 4 mm, with a particle size of 5 μm; Merck, Darmstadt, Germany). The temperature of the column oven was 30 °C. The mobile phase was 0.01 mol/L monopotassium phosphate buffer solution (pH 2.60 adjusted with *o*-phosphoric acid) with isocratic elution at a flow rate of 0.5 mL/min. Identification and quantification of the organic acids were based on the external standards of an organic acid kit (Merck, Darmstadt, Germany)

### 3.12. Phenolics

The quantification of phenolics in noni juice was conducted using the method described by Saikia et al. [43]. An HPLC (Shimadzu LC-10AD, Kyoto, Japan) equipped UV-Vis detector (Shimadzu SPD-20A, Kyoto, Japan) set at 325 nm was used. Exactly 20 μL of the filtered sample was injected into a LiChrospher RP-18 column (125 mm × 4 mm, with a particle size of 5 μm; Merck, Darmstadt, Germany). The mobile phase consisted of acidified ultrapure water (pH 3.2 adjusted with glacial acetic acid) (mobile phase A) and methanol (mobile phase B). The gradient elution parameters were as follow: 20% B (0–8 min), 35% B (9–12 min), 55% B (13–16 min), 70% B (17–20 min), 80% B (21–30), and 90% B (31–34 min), followed by column washing with 35% B (35–39 min) and a final elution with 20% B (40–45 min). The column temperature was kept at 30 °C at a flow rate of 0.5 mL/min. Identification and quantification of the phenolics were based on the external standards of vanillic acid, rutin, quercetin, and scopoletin.

### 3.13. Microbial Analysis

Noni juice was serially diluted with sterile 0.1% peptone water and plated into microbiological media. Aerobic mesophilic bacteria count was examined using plate count agar (PCA), and yeast and mold counts were examined using potato dextrose agar (PDA) combined with 10% tartaric acid. The PCA plate was incubated at 37 °C for 1 day, whereas the PDA plate was incubated at 25 °C for 5 days [26]. Microbial tests were conducted on week 0 (after one day of storage), week 4, and week 8 on juice samples stored under refrigerated (4 °C) conditions. Results were expressed as log colony forming units (CFU) per mL of juice.

### 3.14. Statistical Analysis

All analyses were conducted in triplicate. The data were analyzed using one-way analysis of variance (ANOVA) followed by Tukey’s honestly significant difference (HSD) <post hoc test using IBM SPSS Statistics 26.0 (IBM Corp., New York, NY, USA). The level of significance was set at *p* < 0.05.

## 4. Conclusions

The effects of different sonication durations (20, 40, and 60 min) on the physicochemical properties of noni juice were investigated. Data were compared with fresh and pasteurized juice samples. The titratable acidity, pH, total soluble solids, and total carotenoids were unaffected by sonication and pasteurization. The concentrations of malic and ascorbic acids were improved in the sample sonicated for 60 min. Noni juice sonicated for 60 min resulted in increments of 53% scopoletin, 46% rutin, and 35% vanillic acid compared to the fresh sample. Despite being high in phenolics, organic acids, and antioxidant activity of the S60 sample, the microbial counts showed no difference relative to the fresh sample after 8 weeks of refrigerated storage. Further studies analyzing the influence of storage conditions on the nutritional properties of sonicated noni juice should be conducted.

## Figures and Tables

**Table 1:** Total soluble solids, pH, titratable acidity, viscosity, and color.

Sample	TSS (°Brix)	pH	Titratable Acidity (%)	Viscosity (mPa s)	Color					
					L*	a*	b*	*h°*	C*	Δ*E*
PAS	1.37 ± 0.06 ^a^	3.91 ± 0.01 ^a^	0.18 ± 0.02 ^a^	14.40 ± 0.40 ^a^	47.66 ±0.32 ^a^	−0.41 ± 0.03 ^a^	5.46 ± 0.03 ^bc^	94.33 ± 0.34 ^a^	5.48 ± 0.03 ^bc^	1.16 ± 0.85 ^a^
FRE	1.33 ± 0.06 ^a^	3.90 ± 0.01 ^a^	0.17 ± 0.01 ^a^	16.27 ± 0.23 ^b^	47.55 ± 0.47 ^a^	−0.44 ± 0.03 ^a^	6.47 ± 0.75 ^c^	93.94 ± 0.73 ^a^	6.49 ± 0.74 ^c^	REF
S20	1.30 ± 0.00 ^a^	3.91 ± 0.01 ^a^	0.17 ± 0.01 ^a^	17.07 ± 0.61 ^bc^	52.92 ± 0.31 ^b^	−0.39 ± 0.06 ^a^	4.30 ± 0.36 ^ab^	95.21 ± 0.31 ^a^	4.32 ± 0.37 ^ab^	5.84 ± 0.19 ^b^
S40	1.30 ± 0.00 ^a^	3.91 ± 0.01 ^a^	0.17 ± 0.01 ^a^	17.73 ± 0.23 ^c^	52.35 ± 0.53 ^b^	−0.35 ± 0.11 ^a^	4.59 ± 0.28 ^ab^	94.37 ± 1.54 ^a^	4.60 ± 0.27 ^ab^	5.20 ± 0.61 ^b^
S60	1.33 ± 0.06 ^a^	3.91 ± 0.01 ^a^	0.17 ± 0.01 ^a^	17.33 ± 0.23 ^c^	52.71 ± 0.47 ^b^	−0.35 ± 0.06 ^a^	3.45 ± 0.70 ^a^	95.75 ± 0.16 ^a^	3.47 ± 0.71 ^a^	6.11 ± 0.44 ^b^

TSS: total soluble solids, PAS: pasteurized noni juice, L*: lightness, a*: green (−) or red (+), b*: blue (−) or yellow (+), *h°*: hue, C*: chroma, Δ*E*: total color differences, REF: reference for total color difference calculation, FRE: fresh noni juice, S20: noni juice sonicated for 20 min, S40: noni juice sonicated for 40 min, and S60: noni juice sonicated for 60 min. Different superscript letters (^a^, ^b^, and ^c^) indicate significant differences at *p* < 0.05.

**Table 2:** Total carotenoids, phenolics, and flavonoids.

Sample	TC (mg *β*CE/100 mL)	TPC (mg GAE/100 mL)	TFC (mg RE/100 mL)
PAS	0.01 ± 0.00 ^a^	2.62 ± 0.05 ^a^	1.14 ± 0.17 ^ab^
FRE	0.02 ± 0.00 ^a^	2.93 ± 0.07 ^b^	1.01 ± 0.19 ^a^
S20	0.01 ± 0.00 ^a^	2.88 ± 0.08 ^b^	1.20 ± 0.17 ^ab^
S40	0.01 ± 0.00 ^a^	2.93 ± 0.05 ^b^	1.40 ± 0.06 ^ab^
S60	0.02 ± 0.00 ^a^	3.19 ± 0.06 ^c^	1.48 ± 0.11 ^b^

TC: total carotenoid content, TPC: total phenolic content, TFC: total flavonoid content, PAS: pasteurized noni juice, FRE: fresh noni juice, S20: noni juice sonicated for 20 min, S40: noni juice sonicated for 40 min, and S60: noni juice sonicated for 60 min. Different superscript letters (^a^, ^b^ and ^c^) indicate significant differences at *p* < 0.05.

**Table 3:** Phenolic and organic acid composition.

Sample	Phenolic Compounds (mg/100 mL)				Organic Acids (mg/100 mL)			
	Scopoletin	Rutin	Vanillic Acid	Quercetin	Malic Acid	Fumaric Acid	Citric Acid	Ascorbic Acid
PAS	0.83 ± 0.06 ^a^	2.22 ± 0.25 ^a^	7.95 ± 1.37 ^a^	ND	57.54 ± 3.97 ^a^	0.35 ± 0.05 ^a^	0.90 ± 0.13 ^a^	17.15 ± 0.20 ^a^
FRE	0.96 ± 0.01 ^a^	2.75 ± 0.02 ^a^	9.02 ± 0.25 ^ab^	ND	76.43 ± 2.72 ^b^	0.39 ± 0.05 ^ab^	1.50 ± 0.07 ^b^	26.93 ± 1.17 ^b^
S20	0.98 ± 0.01 ^a^	2.64 ± 0.02 ^a^	9.52 ± 1.10 ^abc^	ND	84.34 ± 6.52 ^bc^	0.41 ± 0.02 ^ab^	1.86 ± 0.12 ^c^	28.01 ± 1.50 ^bc^
S40	1.00 ± 0.01 ^a^	2.92 ± 0.03 ^a^	11.74 ± 1.02 ^bc^	ND	86.58 ± 4.43 ^bc^	0.51 ± 0.02 ^c^	2.24 ± 0.18 ^d^	30.21 ± 0.71 ^cd^
S60	1.47 ± 0.23 ^b^	4.02 ± 0.84 ^b^	12.17 ± 1.18 ^c^	ND	89.31 ± 5.36 ^c^	0.45 ± 0.04 ^bc^	4.78 ± 0.06 ^e^	31.55 ± 1.43 ^d^

PAS: pasteurized noni juice, FRE: fresh noni juice, S20: noni juice sonicated for 20 min, S40: noni juice sonicated for 40 min, S60: noni juice sonicated for 60 min, ND: not detected. Different superscript letters (^a^, ^b^, ^c^, ^d^ and ^e^) indicate significant differences at *p* < 0.05.

**Table 4:** Antioxidant capacity.

Sample	FRAP (μM Fe^2+^/kg)	TEAC (mM TE/kg)
PAS	29.92 ± 2.57 ^a^	15.52 ± 0.31 ^a^
FRE	37.90 ± 4.69 ^ab^	17.65 ± 0.26 ^b^
S20	38.73 ± 5.23 ^ab^	18.32 ± 0.27 ^b^
S40	45.38 ± 0.72 ^b^	19.39 ± 0.26 ^c^
S60	59.63 ± 1.88 ^c^	19.65 ± 0.30 ^c^

PAS: pasteurized noni juice, FRE: fresh noni juice, S20: noni juice sonicated for 20 min, S40: noni juice sonicated for 40 min, S60: noni juice sonicated for 60 min, FRAP: ferric reducing antioxidant power, and TEAC: Trolox equivalent antioxidant capacity. Different superscript letters (^a^, ^b^ and ^c^) indicate significant differences at *p* < 0.05.

**Table 5:** Microbial load of noni juice under refrigerated (4 °C) storage conditions.

Sample	Week 0 (log CFU/mL)		Week 4 (log CFU/mL)		Week 8 (log CFU/mL)	
	AMB	YM	AMB	YM	AMB	YM
FRE	1.71 ± 0.11 ^b^	ND	1.70 ± 0.02 ^b^	ND	1.84 ± 0.14 ^b^	ND
PAS	0.85 ± 0.21 ^a^	ND	0.60 ± 0.00 ^a^	ND	0.69 ± 0.13 ^a^	ND
S60	1.83 ± 0.00 ^b^	ND	1.75 ± 0.05 ^b^	ND	1.78 ± 0.14 ^b^	ND

AMB: aerobic mesophilic bacteria, YM: yeast and mold, ND: not detected, CFU: colony forming unit, PAS: pasteurized noni juice, FRE: fresh noni juice, S60: noni juice sonicated for 60 min. Different superscript letters (^a^ and ^b^) within the same column indicate significant differences at *p* < 0.05.

## Data Availability

Not applicable.
